# Supplementary material for: Effect of Magnetic Heating on Stability of Magnetic Colloids
Source: Nanomaterials (Basel). 2022 Sep 3;12(17):3064. doi: 10.3390/nano12173064 (PMC9458204; doi:10.3390/nano12173064)
Supplement: Supplementary file 1 [file nanomaterials-12-03064-s001.zip › nanomaterials-1844430-supplementary.pdf]

nanomaterials

MDPI

Article

Effect of Magnetic Heating on Stability of Magnetic Colloids

Andrzej Drzewiński *, Maciej Marć, Wiktor W. Wolak and Mirosław R. Dudek

Institute of Physics, University of Zielona Góra, ul. Szafrana 4a, 65-069 Zielona Góra, Poland
* Correspondence: a.drzewinski@if.uz.zgora.pl

S1. Supplementary Materials

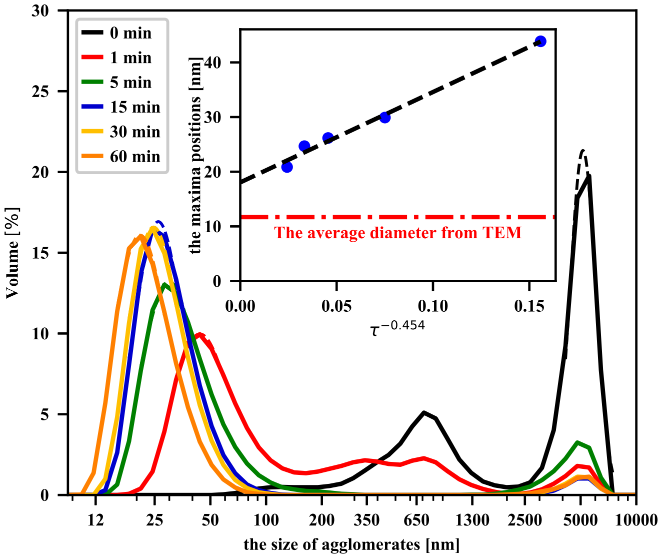

Figure S1 . The volume-based size distribution of the agglomerates of the nanoparticles dispersed in an aqueous suspension with pH = 11.5 for different sonication durations. An interpolation of the distribution function near their maximum is marked with a dashed line. The inset suggests a power-law trend of the dependence of the mean hydrodynamic diameter on the duration of the sonication process, where the HD offset value is 17.8 nm. The dimensionless quantity τ is defined as (the sonication time)/(1 min).

Table S1 . Slopes and SAR values of nanoparticles for sonication durations as in panel (a) in Figure 4.

Sonication Time [min]	Slope [°C/min]	SAR [W/g]
0	1.55	16.61
1	3.09	33.14
5	4.39	47.06
15	5.15	55.19
30	4.87	52.15
60	5.40	57.88
120	5.34	57.24

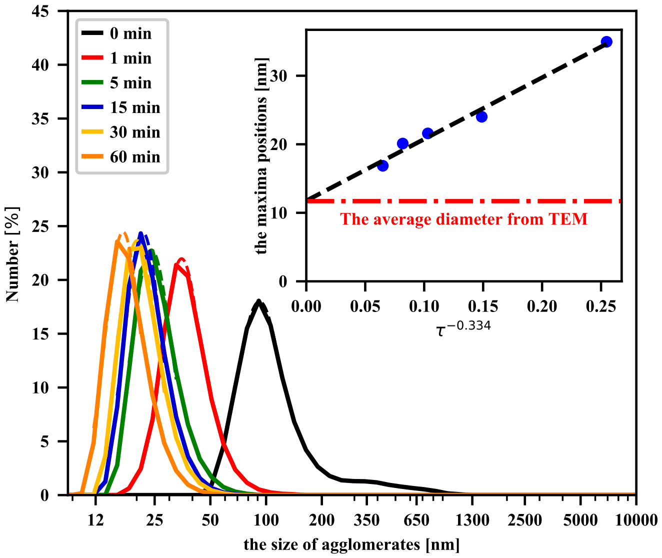

Figure S2 . The number-based size distribution of the agglomerates of the nanoparticles dispersed in an aqueous suspension with pH = 11.5 for different sonication durations. An interpolation of the distribution function near their maximum is marked with a dashed line. The inset suggests a power-law trend of the dependence of the mean hydrodynamic diameter on the duration of the sonication process, where the HD offset value is 11.5 nm. The dimensionless quantity τ is defined as (the sonication time)/(1 min).

Table S2 . SAR values of nanoparticles for sonication durations as in panel (b) in Figure 4. The symbol ΔSAR means the difference between the highest and the lowest value of the SAR coefficient for a given sonication time.

Sonication Time [min]	SAR [W/g]	SAR_{mean} [W/g]	ΔSAR	$\Delta SAR/SAR_{mean}$ [%]
0	25.86<br>20.71<br>21.34<br>22.19<br>25.51	23.12	5.16	22.3
15	48.55<br>43.74<br>47.14<br>45.09<br>43.81	45.67	4.81	10.5
120	51.52<br>53.35<br>53.50<br>55.76	53.53	4.24	7.9
